# Dual-function of Baicalin in nsPEFs-treated Hepatocytes and Hepatocellular Carcinoma cells for Different Death Pathway and Mitochondrial Response

**DOI:** 10.7150/ijms.34876

**Published:** 2019-09-07

**Authors:** Yubo Wang, Shengyong Yin, Yuan Zhou, Wuhua Zhou, Tianchi Chen, Qinchuan Wu, Lin Zhou, Shusen Zheng

**Affiliations:** 1 Department of Hepatobiliary and Pancreatic Surgery, First Affiliated Hospital, Key Laboratory of Combined Multi-Organ Transplantation, Ministry of Public Health, School of Medicine, Zhejiang University, Hangzhou, Zhejiang Province 310003, China; 2 Department of hepatobiliary and pancreatic surgery, Taihe Hospital, Hubei University of Medicine, Hubei, China

**Keywords:** Dual Function, Hepatocellular Carcinoma, Nanosecond pulsed electric fields, Baicalin, Mitochondrial transmembrane potential

## Abstract

Nanosecond pulsed electric fields (nsPEFs) is emerged as a potential curative modality to ablate hepatocellular carcinoma (HCC). The application of local ablation is usually limited by insufficiency of liver function. While baicalin, a flavonoid isolated from Scutellaria baicalensis Georgi, has been proven to possess both anti-tumor and protective effects. Our study aimed to estimate different responses of hepatic cancer cells and hepatocytes to the combination of nsPEFs and baicalin. Cell viability, apoptosis and necrosis, mitochondrial transmembrane potential (MTP) and reactive oxygen species (ROS) were examined by CCK-8, FCM, JC-1 and fluorescent probe, respectively. After treatment by nsPEFs, most hepatocytes died by apoptosis, nevertheless, nearly all cancer cells were killed through necrosis. Low concentration of baicalin synergically enhanced nsPEFs-induced suppression and necrosis of HCC cells, nevertheless, the application of baicalin protected normal hepatocytes from the injury caused by nsPEFs, owing to elevating mitochondrial transmembrane potential and reducing ROS generation. Our work provided an advantageous therapy for HCC through the enhanced combination treatment of nsPEFs and baicalin, with which could improve the tumor-ablation effect and alleviate the injury of hepatic tissues simultaneously.

## Introduction

Liver cancer, of which about 75%-85% cases are hepatocellular carcinoma (HCC), is quite prevalent and lethal worldwide 1. Less than 30% of HCC patients have an opportunity to undergo surgery due to poor physical condition, major vascular invasion or shortage of organ supply. For most cases of HCC, local treatments, comprising trans arterial chemoembolization (TACE), radiofrequency ablation (RFA) and percutaneous ethanol injection (PEI), are widely adopted due to unavailable resection of tumor 2-4. However, these local strategies are frequently limited by multiple complications, for instance, thermal and chemical injuries. To surmount these defects, a novel treatment nanosecond pulsed electric fields (nsPEFs), which employs nanosecond duration electrical pulses with utmost voltage and field strength, has been lately developed to ablate solid tumor by non-thermal way 3. Instantaneous huge power of nsPEFs triggers death of tumor cells but is merely harmful to intrahepatic ducts 5. NsPEFs can induce cell death through several mechanisms, mainly including the reversible electroporation of plasma membrane (PM) and mitochondria damage 6, 7. These high intensity pulses expand the membrane permeability and ultimately permit small molecules to penetrate the plasma membrane such as calcium or dyes, for instance, propidium (PI) and trypan blue (TB)^8^, ^9^. In addition, the latest evidence has shown that the application of nsPEFs with much shorter pulse duration has more impact on intracellular organelle than plasma membrane 10, which leads to the dissipation of mitochondria transmembrane potential 7. Furthermore, nsPEFs can trigger calcium overload 11, stress responses 12, apoptosis 11, 13, 14 and diverse signal kinase pathways activation in cancer cells 15-17, and the ablation effect of nsPEFs has been validated on various malignancies including hepatocellular carcinoma 18, melanoma 19, pancreatic cancer 20, squamous cell carcinoma 21 etc.

Although nsPEFs can effectively ablate hepatic tumors, it is inevitable for nsPEFs to damage normal hepatic tissues, which might cause liver insufficiency. In order to improve the therapeutic effect of nsPEFs, baicalin, the major flavonoid and main active ingredient purified from traditional Chinese medicine *Scutellaria baicalensis Georgi*, whose chemical constitution is known 22, is employed. Baicalin has been reported as an effective agent exhibiting multiple pharmacological functions, for instance, anti-tumor, anti-inflammatory and anti-oxidation 23-25. These pharmacological functions are depend on the arrest of cell cycle, induction of apoptosis, reduction of reactive oxygen species (ROS) and stabilization of mitochondrial transmembrane potential (MTP) 26, 27. Baicalin or baicalein, 90% of which would convert into baicalin in blood, has been reported to be lethal to hepatic tumor by suppressing tumor migration and invasion, inducing apoptosis and inhibiting tumor growth 28, 29.

Since the anti-tumor function of nsPEFs has been validated, we hypothesized that the application of nsPEFs could effectively ablate HCC and the normal hepatic tissue damage within the range of effective electric field could be prevented by agents, such as baicalin. In this study, low concentration of baicalin was used after the application of nsPEFs to enhance the tumor-elimination capability and protect normal hepatocytes from the injury caused by nsPEFs simultaneously. The results demonstrated a dual function that the combined therapy could inhibit HCC cells more effectively by enhancing necrotic cell death but alleviate the damage of normal hepatocytes by preserving mitochondrial transmembrane potential and cleaning up cellular reactive oxygen species. These findings elicited a potential clinical strategy to eliminate hepatocellular carcinoma more sufficiently while alleviating the damage of normal hepatic tissues and provided a conceivable clinical guidance for nsPEFs.

## Materials and Methods

### Cell culture

Human normal hepatocyte line QSG-7701 and human hepatocellular carcinoma cell line MHCC-97H were purchased from the Chinese Academy of Science. High metastatic HCC cell line HCC-LM3 was purchased from the Liver Cancer Institute, Zhongshan Hospital, Fudan University. QSG-7701 cells were maintained in RPMI-1640 (Gibco-Invitrogen, Carlsbad, CA, USA) and MHCC-97H, HCC-LM3 cells were maintained in DMEM (Gibco-Invitrogen, Carlsbad, CA, USA), and both mediums were supplemented with 10% fetal bovine serum (FBS, SAFC Biosciences, Lenexa, KS, USA), 100 unit/ml penicillin and 100 mg/ml streptomycin (SigmaAldrich, St. Louis, MO, USA).

### Isolation and culture of primary mouse hepatocytes

**Figure 1:**
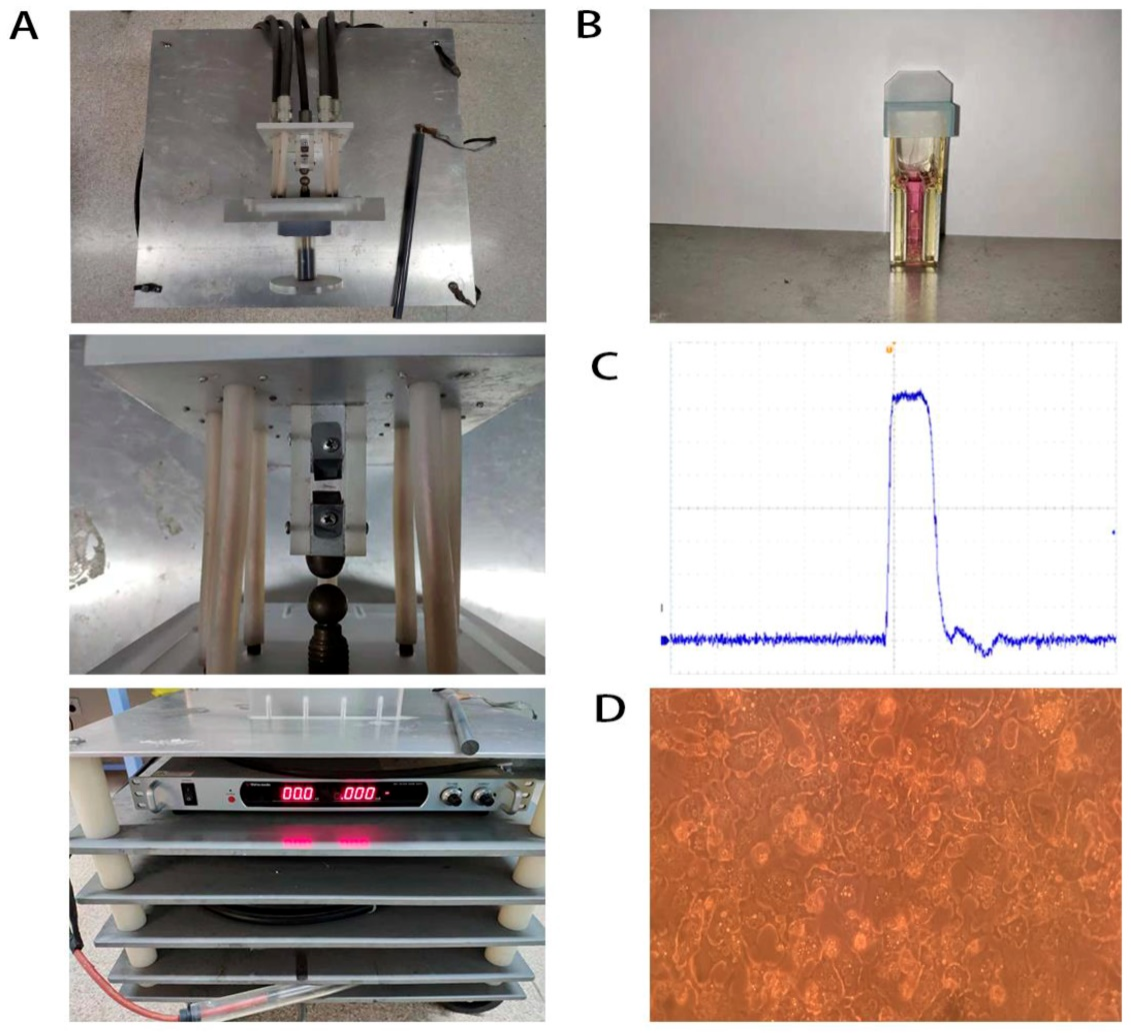
Demonstration of nsPEFs generator (A), cuvettes (B), basic wave of nsPEFs (C) and primary mouse hepatocytes (D).

The primary mouse hepatocytes were isolated from 28-day-old male C57BL/6 mice. The mouse was first anaesthetized and the liver was perfused with Krebs-Ringer buffer and collagenase IV (Sigma Aldrich, St. Louis, MO, USA) without calcium and magnesium. Fibroblasts and liver non-parenchymal cells were removed through DMEM elution. The primary mouse hepatocytes were seeded onto a collagen-coated plate and cultured with the special complete medium of primary mouse hepatocytes (Procell, Wuhan, China) (Figure 1D). All animal experiments were performed in accordance with protocols and regulations of the Experimental Animal Ethics Committee of the First Affiliated Hospital of Zhejiang University (Hangzhou, Zhejiang, China).

### NsPEFs generator and nsPEFs application

NsPEFs generator's essential parameter adjustment was shown in our previous study 35 (Figure 1A). Waveforms were monitored with a digital phosphor oscilloscope (DPO4054, Tektronix, USA, Figure 1C) equipped with a high voltage probe (P6015A, Tektronix, USA). Cells were harvested with trypsin (Gibco-Invitrogen, Carlsbad, CA, USA) and re-suspended in advance preparing medium to a concentration of 2.0×10^6^ cells/ml. Antibiotic free pulse mediums included RPMI-1640 containing 10% FBS for QSG-7701 and DMEM containing 10%FBS for MHCC-97H and HCC-LM3. 1 ml of cell suspension was placed into a 0.4 cm gap cuvette (Biosmith, aluminum plate electrodes, Figure 1B) and exposed to 100ns, 1 HZ, 30, 40, 50, 60, 80 pulses at 15, 25 and 40 kV/cm electric field strength, respectively. These nsPEFs-treated cells (8000 cells/well) were seeded into 96-well plates and incubated for 24h, then their death/viability was detected by CCK-8 (Dojindo, Kumamoto, Japan) assay.

### Baicalin exposure and combination treatment of baicalin and nsPEFs

For baicalin treatment, cells were seeded into 96-well plates and treated by baicalin (Solorbio, Beijing, China) with concentration of 0.1, 1, 10, 20, 40, 80, 160, 320 and 640 μM, respectively, for 24h or 48h, and then their viability was measured with CCK-8 assay. For combined treatment of baicalin and nsPEFs, cells were first exposed to 40P, 15, 25 and 40 kv/cm nsPEFs, then placed into 96-well plates (8000/well). Cells were cultured for 6h to adhere to the plate and then incubated with 0.625μM baicalin for 24h or 48h, following by viability measurement with CCK-8 assay.

### Cell apoptosis and necrosis analysis

Cell apoptosis and necrosis was quantitatively measured using Annexin V-FITC apoptosis detection kit (Dojindo, Kumamoto, Japan) by flow cytometry (FCM). Cells were harvested and washed by PBS, then dyed by FITC and PI (8μl/ml) for 30 min in room temperature before detected by FCM. Double-negative FITC-/PI-, single positive PI+, single positive FITC+ and double-positive FITC+/PI+ represented the living cells, mechanical injury cells, early phase apoptotic cells and late phase apoptotic or necrotic cells, respectively.

### Western-blot assay

RIPA buffer was utilized in lysing cells. All protein concentration was quantified by BCA method. 25μg of proteins from each group were loaded on ExpressPLUSTMPAGE gels (GenScript, USA) and then transferred on PVDF membranes before incubated with primary antibodies (1:2000) overnight. After incubation with HRP-conjugated secondary antibody (1:5000) for 2h, proteins were detected by EZ-ECL (Biological Industries, Israel). Anti-PAR (ab14459), anti-cleaved PARP-1 (ab32561) and anti-β-actin (ab8226) were purchased from Abcam (Cambridge, UK).

### Mitochondrial transmembrane potential measurement

The tetraethylbenzimidazolylcarbocyanine iodide (JC-1) is a cationic dye that accumulates in energized mitochondria. Cells were harvested and washed after handled by different treatment for 24h. For positive control, untreated cells were first mixed with 3μl/ml CCCP and PBS for 1h in 37℃ before mixed with 1μl/ml JC-1 (MultiScience, Hangzhou, China) for 30min in 37℃. For other groups, 1μl/ml JC-1 were mixed with cells and PBS for 30min in 37℃. Eventually, red fluorescence indicated by PE is detected when JC-1 accumulation in mitochondria is sustained by the normal cellular electrochemical potential gradient, or green fluorescence indicated by FITC is present when JC-1 is dispersed into cytoplasm for the dissipation of MTP through FCM.

### Intracellular ROS detection by cell ROS reagent

NsPEFs-treated cells were incubated in the presence or absence of 0.625μM baicalin for 24h, and then washed twice with PBS before stained with 2μl/ml CellROX Green Reagent (ThermoFisher, MA, U.S.A) for 30 min in 37℃ and light-resistant incubator. Ultimately, the concentration of ROS was measured by FCM and fluorescent microscopy, during which the detectable bright green fluorescent signal (at the light wave length of 488nm, or Alexa Fluor 488-A) presented the amount of ROS.

### Cell Viability

Cells were placed into 96-well plates and incubated with 10 μl CCK-8 solution at 37°C. Each sample was replicated 6 times. After 1 hour, the optical density was obtained at 450nm by a spectrophotometer (ELx800; BioTek Instruments, Inc., Vermont, VT, U.S.A). The relative survival rate was calculated by the ratio of OD values of experiment group to OD values of control group.

### Statistical analysis

Raw data were normalized by Microsoft Excel 2010 and figures were generated by GraphPad Prism 5.0 (GraphPad Software, San Diego, CA, U.S.A). Statistical analysis was performed with SPSS 16.0 for windows (SPSS, Chicago, IL, U.S.A). Quantitative variables were expressed as means ± SD. For FCM data, FlowJo V10 (FlowJo LLC, Ashland, OR, U.S.A) was participated. Student's t-test, one-way ANOVA and χ2 analysis were performed to analyze variance. Results were considered statistically significant at P < 0.05. All experiments were repeated three times.

## Results

### Baicalin was more toxic to cancer cells while less toxic to hepatocytes

**Figure 2:**
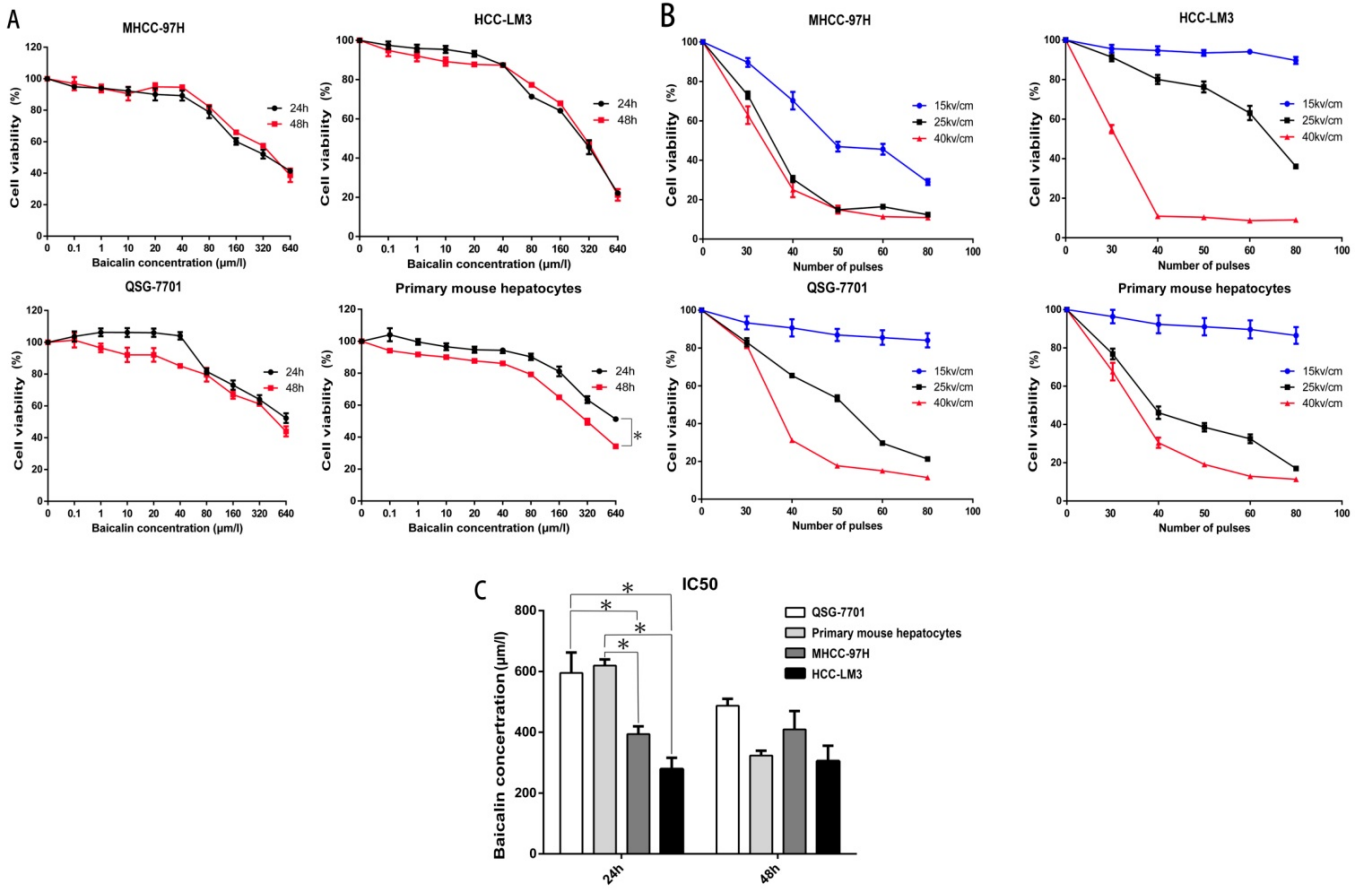
** Effect of Baicalin and nsPEFs on cell viability of HCC cell lines and normal liver cells.** Different cells were treated with baicalin of indicated concentration (A, C) for 24h or 48h and nsPEFs with indicated parameters (B) for 24h. Viabilities were assessed by CCK-8. IC50, half maximal inhibitory concentration. *P<0.05

**Figure 3:**
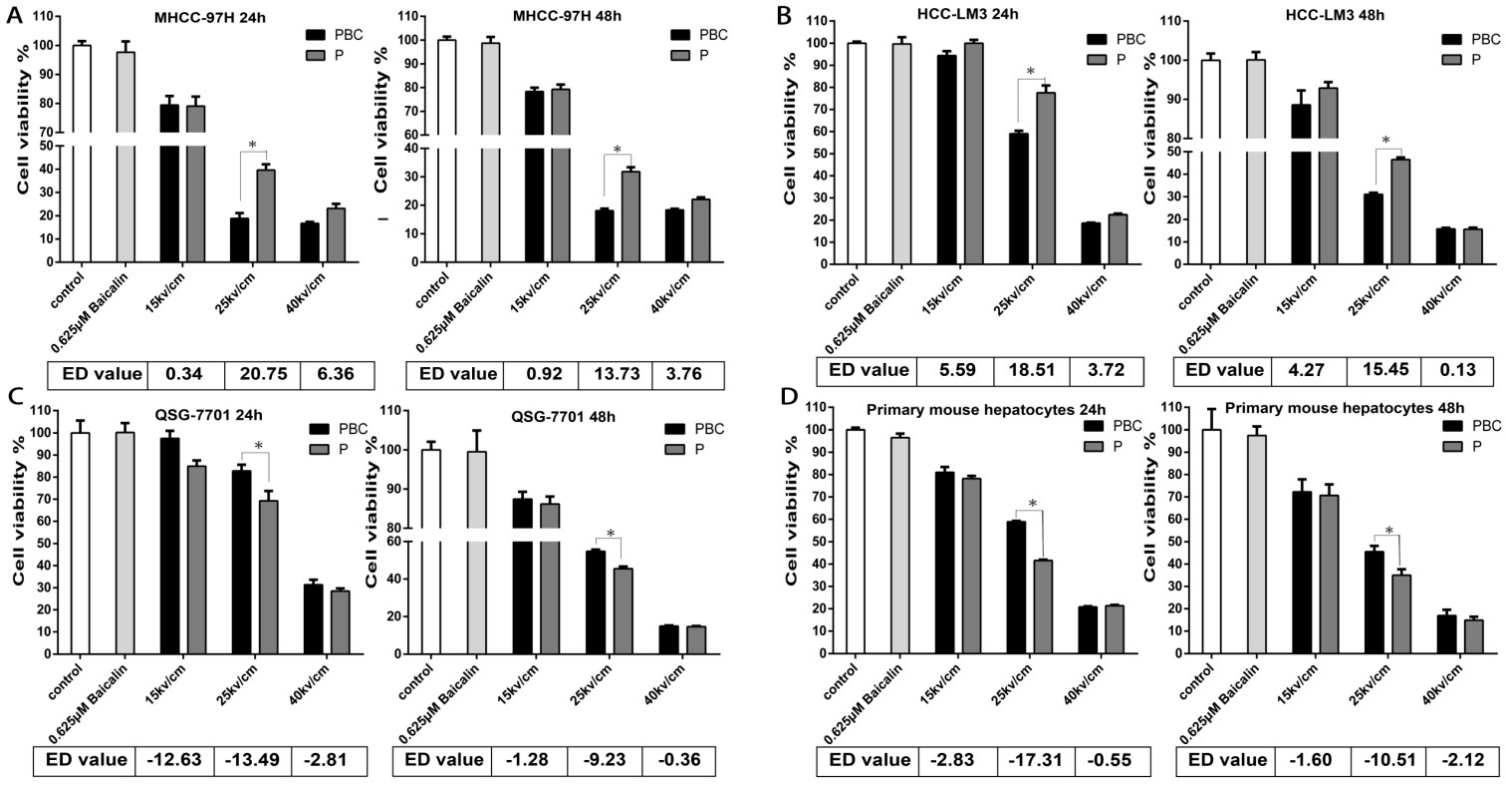
** Synergic effect of treatment of nsPEFs and/or baicalin with low concentration on cell viability.** Treatment of baicalin with concentration of 0.625μM was little harmful to HCC and normal liver cells. HCC cell lines MHCC-97H (A), HCC-LM3 (B) and normal hepatocyte QSG-7701 (C), primary mouse hepatocytes (D) were treated by baicalin with low concentration 0.625μM and/ or nsPEFs with parameter of 40P, 15, 25, 40kv/cm for 24h or 48h, followed by assessment of cell viability. ED value, effect difference value. The ED value were exhibited as mean value of triplicate independent experiments. P, nsPEFs treatment; PBC, combined treatment of nsPEFs and baicalin. *P<0.05

Previous researches have proved that baicalin could suppress HCC cells including HepG2 and SMMC-7721 and has fewer side effects to normal hepatocytes, the Chang liver cell line 26, 28, 29. In our study, we first assessed the toxicity of baicalin on HCC cell lines MHCC-97H and HCC-LM3, normal human hepatic cell line QSG-7701 and primary mouse hepatocytes. The survival rates of all cells are shown in Figure 2A. Although the inhibition was not significantly different at 24h or 48h after baicalin treatment in HCC cell lines, primary mouse hepatocytes were more sensitive to baicalin at 48h after treatment (P<0.05). In order to reduce the toxicity of baicalin on normal hepatocytes, 24h after baicalin treatment was chosen in the following mechanism researches. This conclusion will be further corroborated in Figure 3. IC_50_ values of HCC cells was much higher than normal hepatocytes after 24h while no statistical differences in IC_50_ values between HCC cells and normal hepatocytes after 48h (Figure 2C). Observations of differences between HCC cells and normal hepatocytes in IC_50_ values demonstrated that baicalin was more toxic to cancer cells than normal hepatocytes at 24h after baicalin treatment.

### Determination of appropriate nsPEFs parameters

To determine the appropriate parameters of nsPEFs for the combined treatment, a train of nsPEFs with the doses of 30, 40, 50, 60, 80 in pulse number and the strength of 15kv/cm, 25kv/cm, 40kv/cm in electric fields were applied. The reason why we selected 30-80 in pulse number was the generator was set to generate 10 pulses as a group in order to stabilize the wave form and reduce pulse stretching. As shown in Figure 2B, the number of pulses was chosen at 40 pulses because the cell viability after nsPEFs treatment with 40kv/cm at more than 40 pulses in HCC-LM3 cell line was too low (about 10%) and that with 25kv/cm and 40kv/cm at 30 pulses in QSG-7701 cell line had no statistical difference. Interestingly, although nsPEFs non-selectively killed cells, the tolerance of different cells to nsPEFs was in variety. HCC cell line MHCC-97H seemed to be more susceptible to nsPEFs, while other cells exhibited no apparent difference, which indicated different cell structure might correlate with the different susceptibility to nsPEFs.

### Combined treatment of nsPEFs and baicalin synergistically inhibited HCC cells whereas protected normal hepatocytes

After the treatment of 40P, 25kv/cm nsPEFs combined with a train of baicalin with the concentration of 0-80μM on all cells within 24h or 48h, it appeared that the concentration of 0.625μM was more appropriate for baicalin to alleviate the injury of nsPEFs on normal hepatocytes and enhance the suppression of nsPEFs on HCC cells simultaneously (Figure S1). To further explore the synergistic function of combined application of nsPEFs and baicalin, all cells were treated with 0.625μM baicalin and nsPEFs with 40 pulses and 15, 25, 40 kv/cm electric field strength for 24h and 48h. In this regarding experiment, effect difference (ED) value is assessed according to the relative cell viability of the group with nsPEFs treatment alone to that of the group with combined treatment, and the positive or negative value represented synergistic lethal or protective effects, respectively. Figure 3 shows that treatment with 0.625μM baicalin alone had no impact on cell viability within both cancer cells and hepatocytes. However, combined treatment of this relative low concentration of baicalin and nsPEFs with 40 pulses, 25kv/cm caused striking inhibition on both MHCC-97H and HCC-LM3 cell lines compared with the treatment of nsPEFs alone, with the ED value of 20.75, 18.51 at 24h, respectively and 13.73, 15.45 at 48h, respectively (Figure 3A-B). Of interest, combined treatment of 0.625μM baicalin and nsPEFs with 40 pulses, a train of 15-40 kv/cm field strength for 24h and 48h lead to negative ED values within QSG-7701 cell line and primary mouse hepatocytes (Figure 3C-D). The absolute value of ED on cell line MHCC-97H, HCC-LM3, QSG-7701 and primary mouse hepatocytes at 24h were higher than that at 48h, which indicated that the synergism of combined treatment at 24h was more powerful than 48h and further confirmed the conclusion above that 24h was the more appropriate time point. These observations suggested completely different lethal mechanisms caused by nsPEFs between HCC cells and normal hepatocytes.

### NsPEFs was able to trigger distinct cell death modes

**Figure 4:**
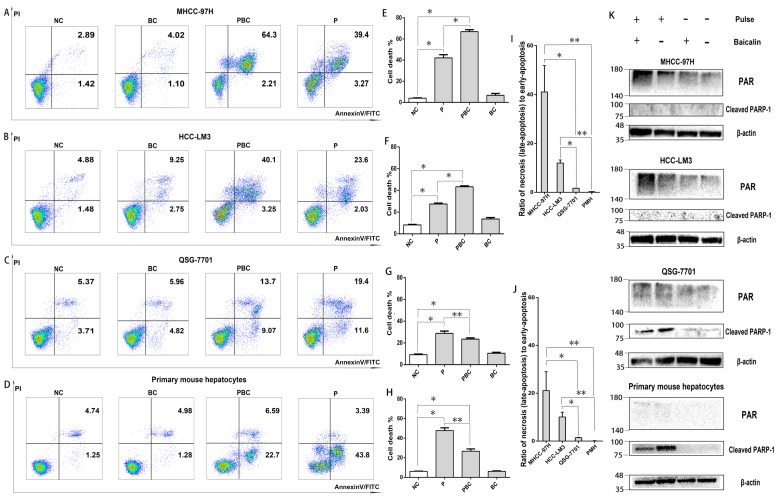
** Baicalin enhanced cell necrosis but inhibited apoptosis under nsPEFs treatment.** Cell death of MHCC-97H, HCC-LM3, QSG-7701 cell lines and primary mouse hepatocytes after treatment of baicalin and/ or nsPEFs for 24h was evaluated by flow cytometry (A-D). The percentage of cell death (E-H) or ratio of necrosis (or late apoptosis) to early apoptosis for the group with nsPEFs treatment alone (J) and the group with combined treatment (I) were quantified according to flow cytometry results. The amount of cell death markers, PAR and cleaved PARP-1, were examined by western-blot assay (K). PMH: primary mouse hepatocytes; NC, cells without any treatment of nsPEFs or baicalin as negative control; P, nsPEFs treatment; BC, baicalin treatment; PBC, combined treatment of nsPEFs and baicalin. *P<0.05, **P<0.01

Since the ED value of 15kv/cm and 40kv/cm on all cells were too small and had no statistical difference, the appropriate electric field of nsPEFs was 25kv/cm. In summary, the right parameter of combined treatment in the following mechanism experiments was 40 pulses, 25kv/cm for nsPEFs and 0.625μM for baicalin within 24h. To explore the underlying mechanisms of lethal and protective effects, FCM was applied to evaluate the effect of treatment of nsPEFs and/or baicalin on cell death. As shown in Figure 4A-H, the cell death (total number of necrosis and apoptosis) triggered by nsPEFs was enhanced by low concentration of baicalin within MHCC-97H and HCC-LM3 cell lines whilst suppressed within QSG-7701 cell line and primary mouse hepatocytes. In addition, the contribution of combined treatment to ratio of necrotic cells to apoptotic cells was facilitated within MHCC-97H and HCC-LM3 cell lines whereas nearly stable within QSG-7701 cell line and primary mouse hepatocytes (Figure 4I-J), reflecting that there were two different death modes between HCC cells and normal hepatocytes.

In order to confirm the potential distinct cell death modes within HCC and normal liver cells, the protein levels of Poly (ADP-ribose) (PAR) and cleaved PAR polymerase-1 (cleaved PARP-1), which indicated necrosis and apoptosis, respectively, were examined by western-blot assay. PAR is polymerized by PARP-1, a downstream target of activated caspase 3, within the nucleus 30, 31, 32. In accordance, PAR amount was significantly increased within MHCC-97H and HCC-LM3 cell lines while hardly detected within QSG-7701 cell line and primary mouse hepatocytes after treatment of nsPEFs or combined treatment of nsPEFs and baicalin (Figure 4K). Moreover, the protein expression of cleaved PARP-1 was hardly examined within MHCC-97H and HCC-LM3 cell lines while remarkably elevated within QSG-7701 cell line and primary mouse hepatocytes after treatment of nsPEFs or combined treatment of nsPEFs and baicalin. Besides, the protein expression of PAR within HCC cells and cleaved PARP-1 within normal hepatocytes were higher and lower, respectively, after combined treatment than nsPEFs treatment alone. Together, above observations mirrored that baicalin treatment was capable of increasing the number of necrotic cells within MHCC-97H and HCC-LM3 cell lines whereas reducing the number of apoptotic cells within QSG-7701 cell line and primary mouse hepatocytes, under the context of nsPEFs treatment. These findings further suggested that, after the treatment of nsPEFs, HCC cell lines and normal hepatocytes exploited two distinct cell death modes, necrosis and apoptosis, respectively, which could be promoted and inhibited by baicalin, respectively.

### Baicalin suppressed nsPEFs-induced mitochondrial transmembrane potential dissipation within normal hepatocytes rather than HCC cell lines

**Figure 5:**
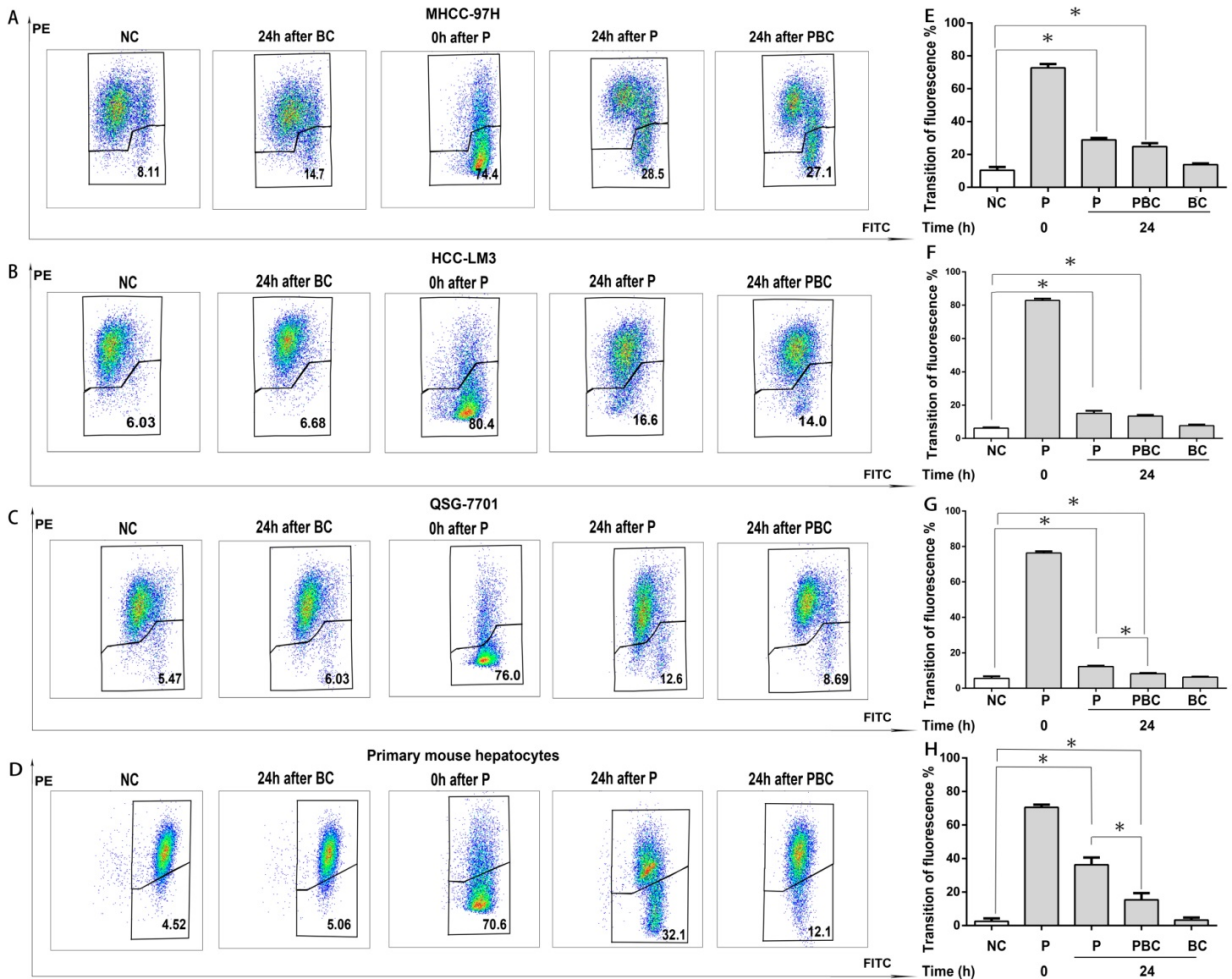
** Different functions of baicalin on nsPEFs-mediated dissipation of mitochondria transmembrane potential between normal hepatocytes and HCC cell lines.** Both HCC cell lines MHCC-97H, HCC-LM3 and normal hepatocytes QSG-7701, primary mouse hepatocytes were treated by baicalin with concentration 0.625μM and/ or nsPEFs with parameter of 40P, 25kv/cm for indicated time, and then their mitochondria transmembrane potential was detected using JC-1 assay through FCM. PE and FITC represented red and green fluorescent signal detected by FCM, respectively (A-D). The quantification of transition of red fluorescent signal to green fluorescent signal (E-H). NC, cells without any treatment of nsPEFs or baicalin as negative control; P, nsPEFs treatment; BC, baicalin treatment; PBC, combined treatment of nsPEFs and baicalin. *P<0.05

It has been evident that nsPEFs is able to dissipate MTP and finally result in cell apoptosis 7 while baicalin is protective to injured cells 27. Therefore, it was reasonable for baicalin to inhibit nsPEFs-mediated cell apoptosis in normal hepatocytes QSG-7701 and primary mouse hepatocytes through reducing MTP dissipation, but not in HCC cell lines MHCC-97H and HCC-LM3. To testify this hypothesis, the alteration of MTP was examined by cationic lipophilic dye JC-1 within HCC cells and normal hepatocytes after nsPEFs treatment or combined treatment of nsPEFs and baicalin. As depicted in Figure 5, the transition of red to green fluorescent signal instantaneously (at 0h) increased in large extent, but decreased in 24h within both HCC cells and normal hepatocytes after nsPEFs treatment. Furthermore, application of baicalin could significantly enhance the process of decrease in transition of red to green fluorescent signal within primary mouse hepatocytes and cell line QSG-7701 rather than HCC cell lines MHCC-97H and HCC-LM3 at 24h. These results demonstrated that the treatment of low concentration of baicalin was able to suppress MTP dissipation triggered by nsPEFs application within normal hepatocytes QSG-7701 and primary mouse hepatocytes but not HCC cell lines MHCC-97H and HCC-LM3.

### Baicalin cleared up generation of reactive oxygen species (ROS) after nsPEFs treatment

**Figure 6:**
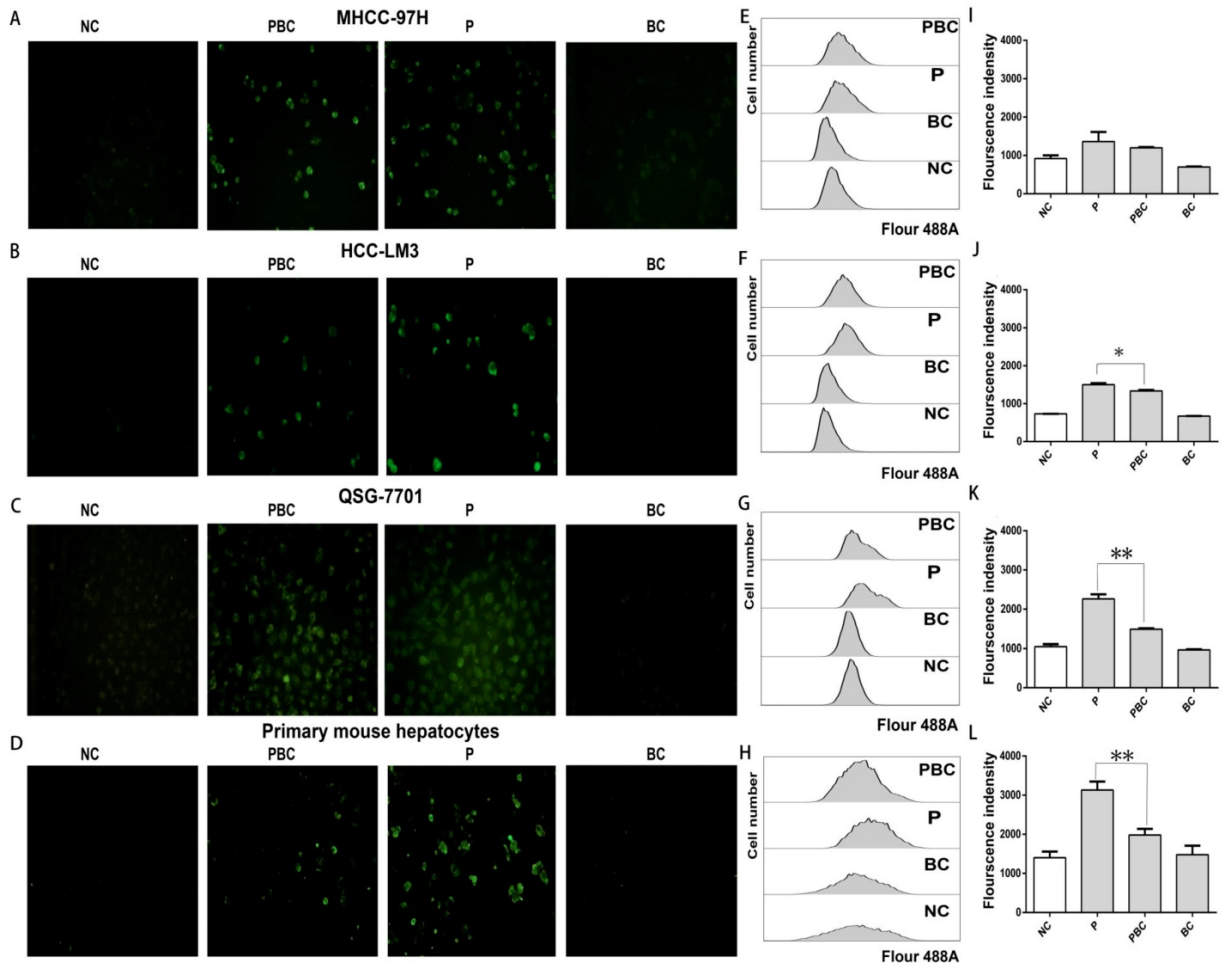
** Baicalin cleared up nsPEFs-induced ROS accumulation within normal hepatocyte.** HCC cell lines MHCC-97H, HCC-LM3 and normal hepatocyte QSG-7701, primary mouse hepatocytes were treated by baicalin with concentration 0.625μM and/ or nsPEFs with parameter of 40P, 25kv/cm for indicated time, and then their ROS production was detected by CellROX Green Reagent through fluorescent microscopy (A-D) or FCM (E-H). Transition of red fluorescent signal to green fluorescent signal was the quantified (I-L). NC, cells without any treatment of nsPEFs or baicalin as negative control; P, nsPEFs treatment; BC, baicalin treatment; PBC, combined treatment of nsPEFs and baicalin. *P<0.05 **P<0.01

Since nsPEFs treatment was capable of triggering accumulation of ROS which could be cleared up by baicalin 33, 34, combined treatment of baicalin probably enabled normal hepatocytes to overcome the oxidative stress caused by nsPEFs and finally to escape from apoptosis. To exactly detect the change of ROS amount after the treatment of nsPEFs or combination of nsPEFs and baicalin, a novel fluorogenic probe for ROS detection which could bind to intracellular DNA as well was utilized. As expected, nsPEFs treatment could significantly promoted ROS production, indicated by dramatic enhancement of green fluorescent signal, which showed the decreasing trend after combined application of nsPEFs and baicalin, especially within normal hepatocytes QSG-7701 and primary mouse hepatocytes (Figure 6A-D). These observations were further confirmed by FCM results (Figure 6E-L), reflecting that baicalin was able to clear up intracellular ROS accumulation to some extent, in particular within normal hepatocytes.

## Discussion

NsPEFs is capable of ablating malignancies through various mechanisms, including inducing apoptosis, increasing PM permeability, activating several kinase pathways, etc.^11^-^17^, ^19^, ^35^-^38^ NsPEFs could efficiently ablate tumor lesions through the low-thermal effect 39, but still along with challenges including tumor recurrence or incidences of injury or inflammation within normal tissues 18, 40. The current study demonstrated that nsPEFs was able to dramatically kill HCC cells and normal hepatocytes at the same parameters. In addition, a novel strategy of combined treatment with nsPEFs and low concentration of baicalin displayed an inspiring therapeutic outcome that facilitated the suppression of HCC cells whereas attenuated injury of normal hepatocytes through reducing the nsPEFs-triggered MTP dissipation and ROS accumulation.

NsPEFs treatment could cause several cell death modes, consisting of apoptosis, autophagy related apoptosis or necrosis, which were largely dependent on cell types, culture status or nsPEFs parameters 41, 42. Despite that, few evidences clarify the difference of cell death modes implemented by different cell lines under the treatment of nsPEFs with the same parameter 43. Here, two distinct cell death modes were uncovered within HCC cell lines and normal hepatocytes. Both results of FCM and protein amount of death markers by immunoblotting showed that cell death of HCC cell lines and normal hepatocytes mainly relied on necrosis and apoptosis, respectively. Specifically, the characteristics of dead HCC cells were double positive PI(+)FITC(+) by FCM and increase in protein amount of PAR by immunoblot, differing from single positive FITC(+) and increased protein level of cleaved PARP-1 by immunoblot within normal hepatocytes. Similarly, dependence on necrosis of malignant cells after nsPEFs treatment was unveiled in the study of nsPEFs treatment on lymphadenoma cell line U937 41. However, another inconsistent evidence revealed that melanoma cancer cells are destructed by nsPEFs through apoptosis 19. This discrepancy might be caused by the different parameters of diverse nsPEFs generator, for instance, pulse width, electric fields strength, pulse stretching or pulse frequency etc. Furthermore, it remained further confirmation that these distinct death modes also existed in malignant and normal cells originated from other organs.

The dual-function of baicalin on anti-tumor and anti-oxidation 23, 29 clued that baicalin might be an ideal pharmacological agent for enhancing ablation effect of tumor and alleviating complications by nsPEFs treatment. Of interest, combined treatment of low concentration of baicalin exhibited the ability to promote the death of HCC cells and reduce the death of normal hepatocytes caused by nsPEFs treatment. Sole application of baicalin with low concentration was quite safe for cell survival (Figure 2-3), differing from killing effects of baicalin with a higher concentration on tumor cells reliable on apoptosis pathway 28, 29. However, treatment of baicalin with higher concentration would be harmful to the survival of normal hepatocytes, which could possibly be accountable for the priority of cell death pathways over anti-oxidation or cell survival protection pathways. Due to distinct cell death modes induced by nsPEFs, necrosis within HCC cells and apoptosis within normal hepatocytes, difference in loss of plasma membrane integrity would probably allow different intracellular accumulation of agents 41, such as baicalin, and eventually affect the fate of different cells.

Intracellular ROS accumulation and MTP dissipation, two typical features of cells after treatment of nsPEFs, play key role in cell death 35. This was substantiated by our findings including spontaneous ROS production with a large amount and MTP dissipation with different degrees within both HCC and normal liver cells. Given roles in anti-oxidant stress 22, 23 and the close correlation with ROS, MTP and apoptosis 44-46, baicalin possesses the potential to clear up nsPEFs-induced intracellular ROS accumulation and stabilize MTP and ultimately overcomes cell death of normal hepatocytes under nsPEFs treatment. As expected, our results demonstrated that combined usage of baicalin with low concentration suppressed the dissipation of MTP and reduced the accumulation of ROS within normal hepatocytes QSG-7701 and primary mouse hepatocytes after the application of nsPEFs, in agreement with the effect of baicalin on the reverse of ultraviolet radiation-induced oxidative damage 27. However, treatment of baicalin with low concentration could induce a descending trend of nsPEFs-induced ROS generation but not MTP recovery within HCC cell line HCC-LM3, supporting that baicalin could clear up ROS non-selectively 47. Additionally, MTP dissipation within necrotic cells 48, resulting in a decrease of ATP and dysfunction of mitochondria, was tougher to overcome 49. The present observations confirmed that nsPEFs-induced MTP dissipation within necrotic HCC cells was more difficult to recover despite the reduction of ROS accumulation to certain extend within HCC cells by low concentration of baicalin. This could explain that baicalin could not attenuate nsPEFs-induced cell death of HCC cell lines.

**Figure 7:**
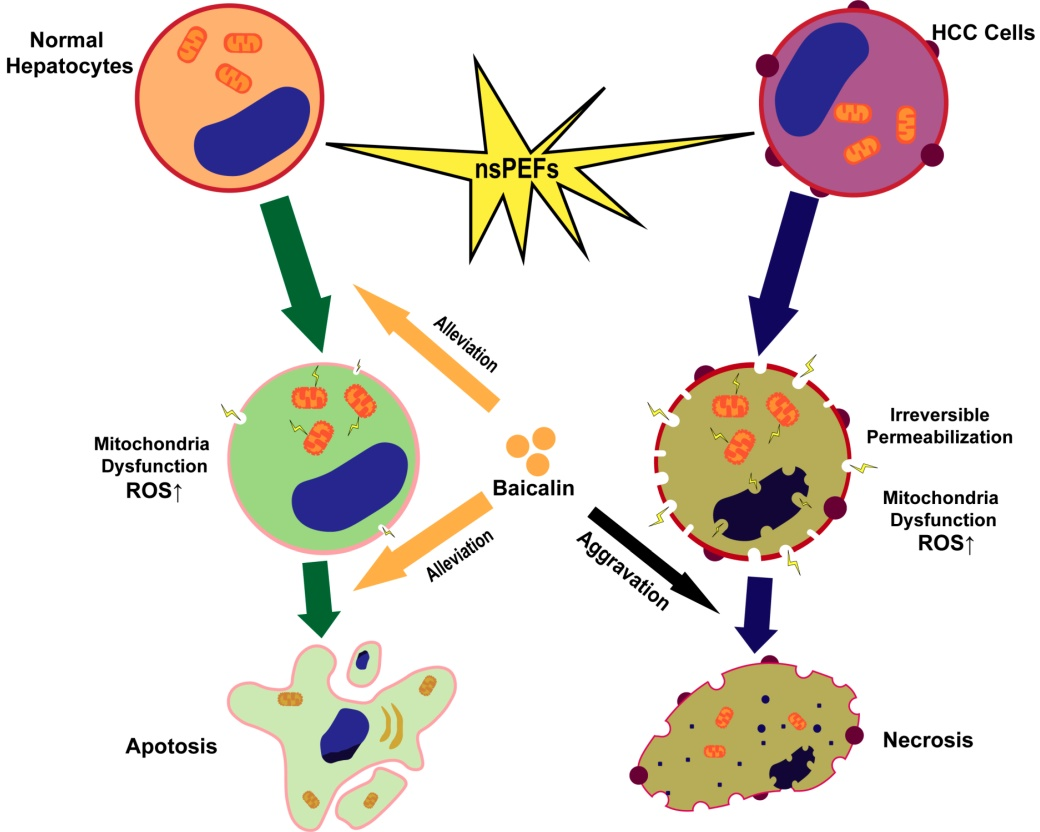
Schematic illustration of mechanism that nsPEFs-triggered cell death was suppressed within normal hepatocytes whilst facilitated within HCC cells by baicalin.

In conclusion, HCC cells and normal hepatocytes are killed by nsPEFs mainly through necrosis and apoptosis, respectively. NsPEFs-induced cell deaths of HCC and normal liver cells can be promoted and attenuated by baicalin with low concentration, respectively, and baicalin prevents normal hepatocytes from damage by nsPEFs largely through clearing up ROS production and stabilizing MTP (Figure 7). Our findings provide an advantageous therapy for HCC that combined treatment of nsPEFs and baicalin could improve the tumor-ablation effect as well as reduce complications of the clinical application of nsPEFs.

## Supplementary Material

Supplementary figure.

## Abbreviations

HCC: hepatocellular carcinoma
nsPEFs: nanosecond pulsed electric fields
ROS: reactive oxygen species
MTP: mitochondrial transmembrane potential
ATP: adenosine triphosphate
PM: plasma membrane
TACE: trans arterial chemoembolization
RFA: radiofrequency ablation
PEI: percutaneous ethanol injection
CCK-8: cell counting kit 8
FCM: flow cytometry
PAR: poly (ADP-ribose)
