# Supplementary material for: Dual-function of Baicalin in nsPEFs-treated Hepatocytes and Hepatocellular Carcinoma cells for Different Death Pathway and Mitochondrial Response
Source: Int J Med Sci. 2019 Sep 7;16(9):1271–82. doi: 10.7150/ijms.34876 (PMC6775272; doi:10.7150/ijms.34876)
Supplement: Supplementary file 1 — Supplementary figure. [file ijmsv16p1271s1.pdf]

Supplementary figure 1: Effect of different concentration of baicalin after nsPEFs treatment on all cells.

HCC cell lines MHCC-97H (A), HCC-LM3 (B) and normal hepatocytes QSG-7701 (C), primary mouse hepatocytes (D) were treated by nsPEFs with the parameter of 40P, 25kv/cm before incubated in baicalin with thse concentration of 0-80μM for 24h and 48h. Cell viability of all cell lines were assessed by CCK-8 assay.

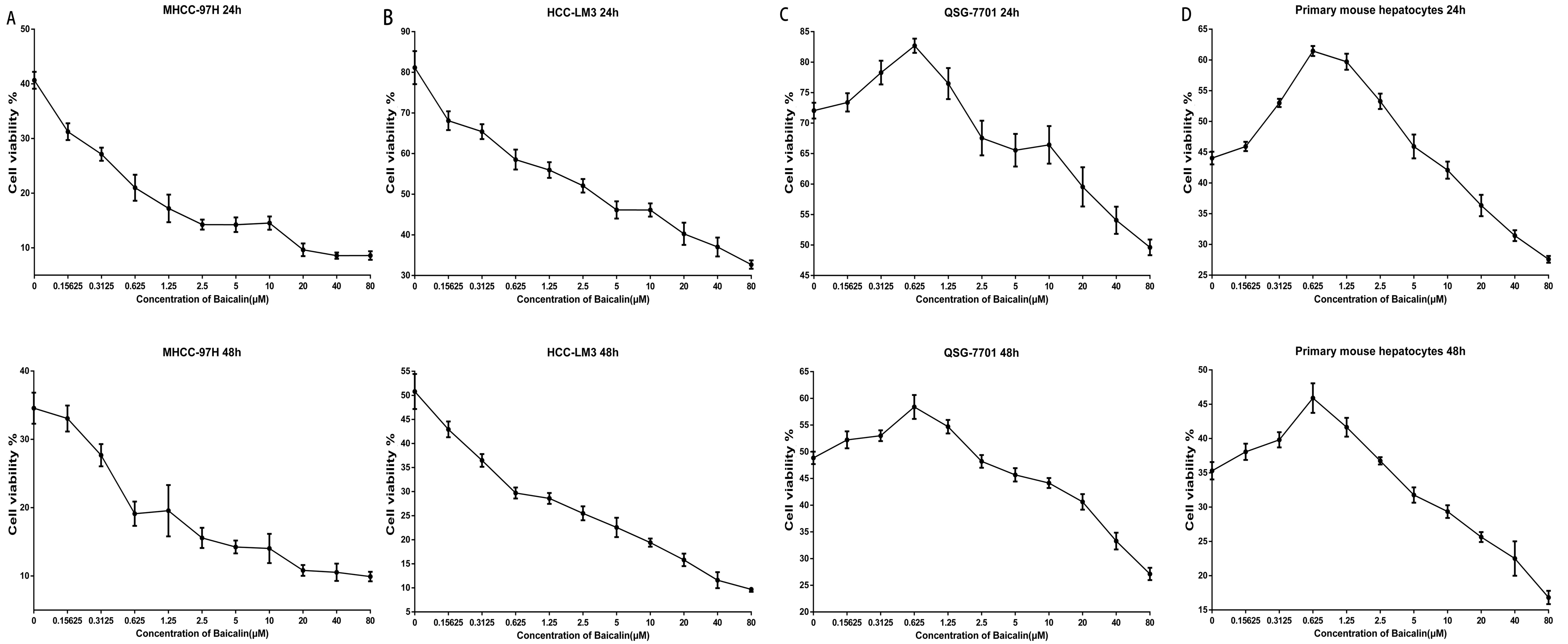

A
MHCC-97H 24h
B
HCC-LM3 24h
C
QSG-7701 24h
D
Primary mouse hepatocytes 24h
MHCC-97H 48h
HCC-LM3 48h
QSG-7701 48h
Primary mouse hepatocytes 48h
Cell viability %
Concentration of Baicalin(μM)
0
0.15625
0.3125
0.625
1.25
2.5
5
10
20
40
80
